# Allosteric Activation of Transglutaminase 2 via Inducing an “Open” Conformation for Osteoblast Differentiation

**DOI:** 10.1002/advs.202206533

**Published:** 2023-04-23

**Authors:** Zhuo Yang, Xiao‐Wen Zhang, Fang‐Fang Zhuo, Ting‐Ting Liu, Qian‐Wei Luo, Yong‐Zhe Zheng, Ling Li, Heng Yang, Yi‐Chi Zhang, Yan‐Hang Wang, Dan Liu, Peng‐Fei Tu, Ke‐Wu Zeng

**Affiliations:** ^1^ State Key Laboratory of Natural and Biomimetic Drugs School of Pharmaceutical Sciences Peking University Beijing 100191 China; ^2^ Proteomics Laboratory Medical and Healthy Analytical Center Peking University Health Science Center Beijing 100191 China

**Keywords:** allosteric activation, forskolin, mitochondria, osteoblast differentiation, transglutaminase 2

## Abstract

Osteoblasts play an important role in the regulation of bone homeostasis throughout life. Thus, the damage of osteoblasts can lead to serious skeletal diseases, highlighting the urgent need for novel pharmacological targets. This study introduces chemical genetics strategy by using small molecule forskolin (FSK) as a probe to explore the druggable targets for osteoporosis. Here, this work reveals that transglutaminase 2 (TGM2) served as a major cellular target of FSK to obviously induce osteoblast differentiation. Then, this work identifies a previously undisclosed allosteric site in the catalytic core of TGM2. In particular, FSK formed multiple hydrogen bonds in a saddle‐like domain to induce an “open” conformation of the *β*‐sandwich domain in TGM2, thereby promoting the substrate protein crosslinks by incorporating polyamine. Furthermore, this work finds that TGM2 interacted with several mitochondrial homeostasis‐associated proteins to improve mitochondrial dynamics and ATP production for osteoblast differentiation. Finally, this work observes that FSK effectively ameliorated osteoporosis in the ovariectomy mice model. Taken together, these findings show a previously undescribed pharmacological allosteric site on TGM2 for osteoporosis treatment, and also provide an available chemical tool for interrogating TGM2 biology and developing bone anabolic agent.

## Graphical abstract

In this study, transglutaminase 2 (TGM2) is identified as a target of forskolin to promote osteoblast differentiation. Forskolin induces an “open” conformation of TGM2 on a previously undisclosed allosteric site, which promotes mitochondrial fusion and ATP production for osteoblast differentiation. These data provide an available chemical tool for interrogating TGM2 and developing anti‐osteoporosis agent.

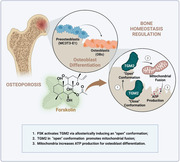

## 1 Introduction

Bone reconstruction is a hot research field in regenerative medicine. The balance between bone resorption and bone formation is crucial for maintaining bone mass and systemic mineral homeostasis.^[^ ^1^ ^]^ Accumulating evidence suggests that the mechanism of bone metabolic regulation is extremely complex, and abnormalities may cause bone metabolic disorders.^[^ ^2^ ^]^

Osteogenic processes are tightly regulated through complex networks activated by distinct osteogenic signaling molecules.^[^ ^3^ ^]^ Transglutaminase 2 (TGM2), is a multifunctional enzyme implicated in the pathogenesis of a number of human diseases, including celiac, neurological, and renal diseases, and various forms of cancer.^[^ ^4^ ^]^ Recently, the biological functions of TGM2 have been extensively studied in matrix maturation and mineralization processes of bone matrix.^[^ ^5^ ^]^ For example, TGM2 is observed to be highly responsible for osteoblast‐like cell differentiation and osteogenic potential.^[^ ^6^ ^]^ Moreover, TGM2 knockout results in lower bone mass and an increased number of osteoclasts.^[^ ^7^ ^]^ Mechanistically, TGM2 contributes to the crosslinking of both intra‐ and extracellular proteins by promoting the formation of *ε*‐(*γ*‐glutamyl) lysine bonds, further leading to the expression of osteogenic genes as well as bone formation and repair.^[^ ^8^ ^]^ Thus, TGM2 targeting may represent a promising strategy to treat osteoporosis by screening specific small molecules to regulate TGM2 biology. However, very few candidates are reported to target TGM2 for osteoblast differentiation and bone reconstruction.

Chemical genetics is a promising strategy for drug‐target prediction.^[^ ^9^ ^]^ Natural products possess diversified biological activities and have become a key resource for drug development.^[^ ^10^ ^]^ Thus, it is worth discovering naturally derived osteogenic compounds to explore their pharmacological targets, thereby clarifying novel osteoblast differentiation mechanisms. Forskolin (FSK) is a unique diterpenoid from the medicinal plant *Coleus forskohlii*.^[^ ^11^ ^]^ FSK has been previously demonstrated to induce the differentiation of adipose‐derived stem cells into Schwann cell‐like cells.^[^ ^12^ ^]^ Meanwhile, FSK also stimulates osteogenic differentiation, suggesting a potential therapeutic effect on bone diseases such as osteoporosis.^[^ ^13^ ^]^

In this study, we synthesized a photoaffinity probe through introducing a diazirine photocrosslinker and a click chemistry handle in FSK. Then, chemical genetics revealed that TGM2 functioned as a direct target protein of FSK by a previously undisclosed allosteric activation mechanism via inducing an “open” conformation of the N‐terminal *β*‐sandwich domain. This resulted in an improvement in mitochondrial energy‐generating processes for osteoblast differentiation. Taken together, we elucidate a druggable hotspot in TGM2 for promoting osteoblast differentiation, and also highlight a previously unknown allosteric activation role for TGM2 as well as potential translational value in the development of bone anabolic agent.

## 2 Results

### 2.1 FSK Promotes the Osteoblastic Differentiation of MC3T3‐E1 Cells

**Figure 1:**
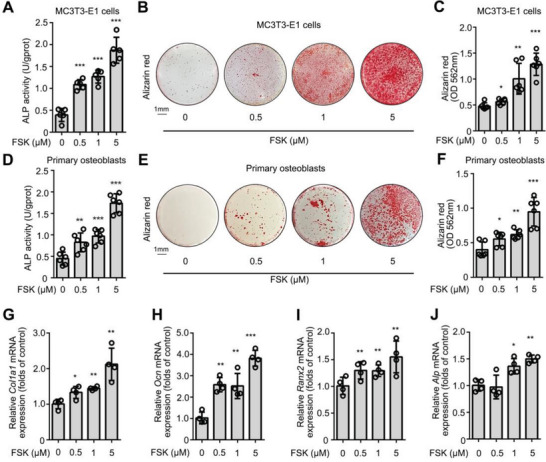
FSK promotes the osteoblastic differentiation of MC3T3‐E1 cells and primary osteoblasts. A) FSK increased the ALP activity of MC3T3‐E1 cells. B,C) FSK upregulated the mineralization of MC3T3‐E1 cells by Alizarin red staining and quantification. D) FSK increased the ALP activity of primary osteoblasts. E,F) FSK upregulated the mineralization of primary osteoblasts by Alizarin red staining and quantification. G‐J) FSK upregulated the expression of osteoblasts differentiation marker genes in MC3T3‐E1 cells. Data are expressed as mean ± SEM for 3–6 individual experiments. ***p < 0.001, **p < 0.01, *p < 0.05 versus control group.

Osteoblast differentiation is an effective strategy to promote bone formation. Thus, we investigated the ability of FSK to promote osteogenic differentiation in pre‐osteoblastic MC3T3‐E1 cells. Alkaline phosphatase (ALP) is a crucial osteoblastic differentiation biomarker that is highly associated with osteogenesis.^[^ ^14^ ^]^ In MC3T3‐E1 cells, we observed that FSK (0.5, 1, and 5 µm) significantly increased ALP activity (**Figure** **1**A) during 7 days of treatment. Moreover, the mineralization assay by Alizarin red staining indicated that FSK (0.5, 1, and 5 µm) obviously increased the mineralization node area and the absorbance of the alizarin red dye in MC3T3‐E1 cells after 21 days of treatment (Figure 1B,C). Furthermore, we confirmed these observations in primary osteoblasts from mouse skulls. Consistent with MC3T3‐E1 cells, FSK (0.5, 1, and 5 µm) significantly increased ALP activity (Figure 1D) and mineralization nodes (Figure 1E,F) in primary osteoblasts. Meanwhile, quantitative real‐time PCR (Rt‐PCR) demonstrated that FSK (0.5, 1, and 5 µm) upregulated the expression of several osteogenesis markers, including *Ocn, Ranx2, Alp*, and *Col1a1* ^[^ ^15^ ^]^ (Figure 1G–J). In addition, FSK slightly increased the viability of MC3T3‐E1 cells, indicating no obvious cytotoxicity (Figure S5A, Supporting Information). Taken together, these results reveal that FSK promotes osteoblastic differentiation and accelerates bone mineralization.

### 2.2 TGM2 Serves as a Cellular Target of FSK

**Figure 2:**
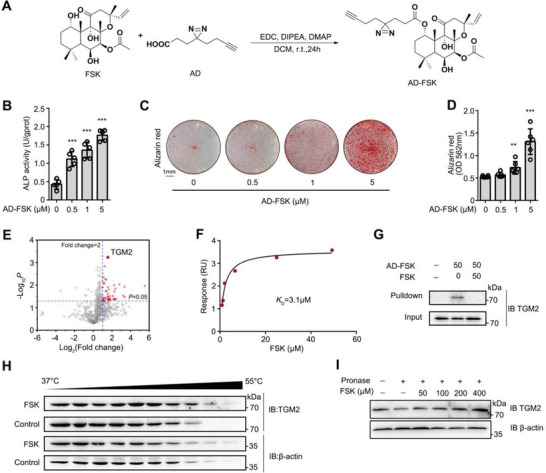
TGM2 serves as a cellular target of FSK. A) Schematic representation of the AD‐FSK synthesis. B,C) AD‐FSK upregulated the mineralization of MC3T3‐E1 cells by Alizarin red staining and quantification. D) AD‐FSK increased the ALP activity of primary osteoblasts. E) Volcano plot of the cellular targets of FSK. F) SPR analysis of FSK‐binding to TGM2. G) Pull‐down analysis of FSK‐binding to TGM2 in MC3T3‐E1 cells. H) FSK promoted resistance of TGM2 to different temperature gradients (CETSA). I) FSK promoted resistance of TGM2 to proteases (DARTS). Data are expressed as mean ± SEM for 3–4 individual experiments. ***p < 0.001, **p < 0.01, *p < 0.05 versus control group.

To identify the cellular target of FSK, we synthesized a FSK molecular probe (AD‐FSK) by introducing a difunctional side chain (AD) with a diazirine and an alkyne (**Figure** **2**A). Diazirine was used for photoaffinity labeling of target proteins in situ by forming covalent bonds. Meanwhile, the alkyne was conjugated with the reporter biotin‐PEG‐azide or 5‐TAMRA‐azide via a click reaction. The chemical structure of AD‐FSK was verified by ^1^HNMR, ^13^CNMR spectra and high‐resolution mass spectrometry (Figures S1–S3, Supporting Information), which suggested that the difunctional side chain was attached to the hydroxyl on the C1 site in FSK skeleton (Figure S4, Supporting Information). To verify the biological activity of AD‐FSK, ALP activity was tested by AD‐FSK treatment and the result demonstrated that AD‐FSK significantly upregulated ALP activity in MC3T3‐E1 cells (Figure 2B). Meanwhile, AD‐FSK showed a mineralization promotion effect similar to that of FSK in MC3T3‐E1 cells (Figure 2C,D). Therefore, these results indicate that AD‐FSK possesses similar biological activity to FSK and is suitable for target identification.

Next, we conducted in‐gel fluorescence analysis to determine the protein labeling capacity of AD‐FSK. MC3T3‐E1 cells were incubated with AD‐FSK and exposed to UV irradiation. The cell lysates were then conjugated to 5‐TAMRA‐azide via click reaction. In‐gel fluorescence scanning showed that the fluorescence intensity of labeled proteins was significantly blocked with an excess amount of FSK for competition (Figure S5B, Supporting Information), indicating that AD‐FSK can be used to capture the protein targets in cells. Next, MC3T3‐E1 cells were treated with AD‐FSK in the presence or absence of FSK. After UV irradiation, cell lysates were prepared and biotin‐PEG‐azide was conjugated to AD‐FSK via a click reaction. Then, the AD‐FSK‐captured proteins were pulled down by streptavidin beads for liquid chromatography tandem‐mass spectrometry (LC‒MS/MS) analysis. A total of 821 proteins were identified, and transglutaminase 2 (TGM2) possessed the highest significance of differences under the competition of FSK (Figure 2E). In particular, previous reports indicated that TGM2 is highly associated with bone formation and repair;^[^ ^16^ ^]^ thus, we speculated that TGM2 may serve as a cellular target of FSK to promote osteogenic differentiation. Furthermore, surface plasmon resonance (SPR) analysis indicated that FSK directly bound to TGM2 (*K*_D_ = 3.1 µm, Figure 2F). We also observed that TGM2 was pulled down by AD‐FSK, which was obviously blocked by an excess amount of FSK for competition (Figure 2G). Meanwhile, a cellular thermal shift assay (CETSA)^[^ ^17^ ^]^ revealed that FSK enhanced the thermal stability of TGM2 (Figure 2H). Drug affinity responsive target stability (DARTS)^[^ ^18^ ^]^ experiments indicated that FSK stabilized TGM2 by increasing resistance to proteolysis (Figure 2I). Collectively, these results demonstrate that FSK directly binds to TGM2 in MC3T3‐E1 cells.

### 2.3 FSK Binds to TGM2 in the Catalytic Core Domain

**Figure 3:**
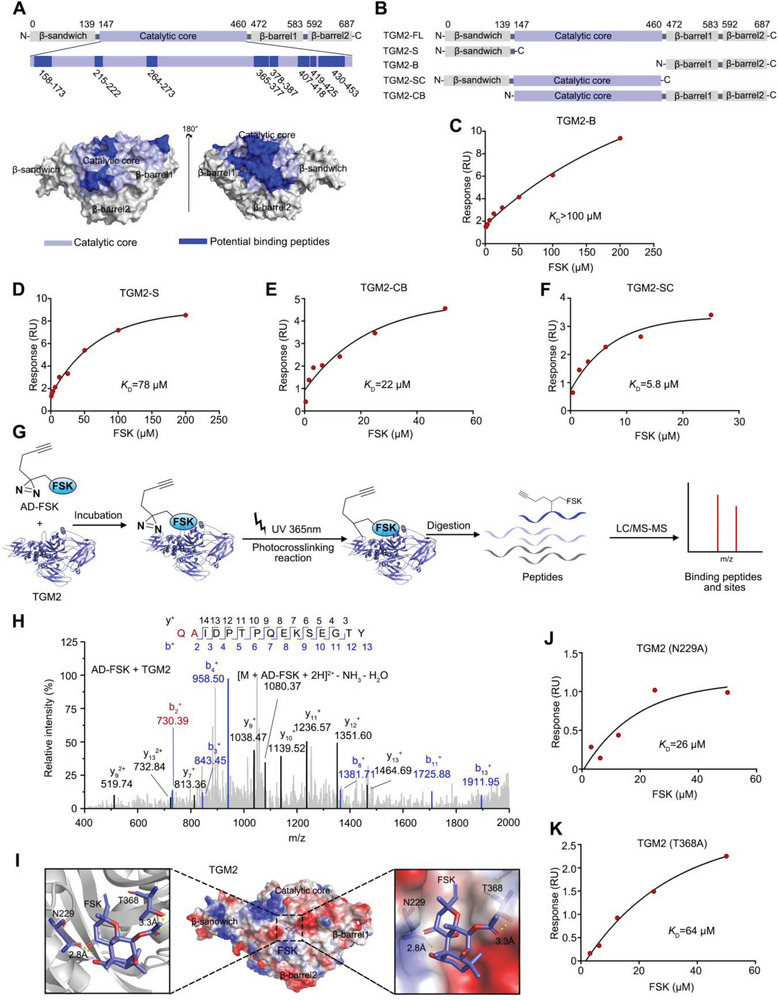
FSK binds to TGM2 in the catalytic core domain. A) Mapping of FSK potential binding peptides to TGM2. B) Diagram of the establishment of four TGM2 truncations. C–F) SPR analysis of the binding ability of FSK with TGM2 truncations. G) Schematic diagram of capturing photoaffinity labeling peptide by AD‐FSK. H) LC‐MS/MS analysis of photoaffinity labeling peptides by AD‐FSK in TGM2. I) Docking model of FSK into the structure of TGM2 (PDB: 1KV3). Hydrogen bonds are shown as yellow dashed lines (N229, T368). J,K) SPR analysis for the binding of FSK with TGM2 N229A and TGM2 T368A mutants.

To determine the FSK‐binding region in TGM2, TGM2 protein was incubated with FSK, followed by mild proteolysis. Given that the FSK‐binding region was more stabilized, the residual peptides (non‐enzymatic hydrolysis) protected by FSK were then identified by LC‒MS/MS analysis. As a result, we determined 11 potential FSK‐protecting peptides, 8 of which were located in the catalytic core domain of TGM2. Of note, the 3D model of TGM2 showed that these peptides were spatially adjacent (**Figure** **3**A), indicating that FSK‐binding region was mainly located in the TGM2 catalytic core domain. To further corroborate these observations, we established four TGM2 truncations based on different domain deletions^[^ ^19^ ^]^ (Figure 3B). SPR analysis demonstrated that FSK did not bind to C‐terminal *β*‐barrel domain truncation (TGM2‐B) (*K*_D_ > 100 µm). Meanwhile, the binding ability of FSK with N‐terminal *β*‐sandwich domain truncation (TGM2‐S) (*K*_D_ = 78 µm), catalytic core domain fusion with C‐terminal *β*‐barrel domain (TGM2‐CB) (*K*_D_ = 22 µm), and catalytic core domain fusion with N‐terminal *β*‐sandwich domain (TGM2‐SC) (*K*_D_ = 5.8 µm) increased successively (Figure 3C,F). Thus, FSK showed a strong binding ability to TGM2‐SC and a moderate binding ability to TGM2‐CB and TGM2‐S, indicating that FSK may prefer to bind to TGM2 in the catalytic core domain. Thus, these experiments provided valuable evidence to further explore the FSK‐binding sites by exact analytical method.

Next, to identify the detailed FSK‐binding sites in TGM2, AD‐FSK was incubated with TGM2 protein for UV irradiation. Then, AD‐FSK was covalently crosslinked with TGM2 at the binding sites by photoaffinity diazirine (Figure 3G). The AD‐FSK‐TGM2 complex was further determined by LC‒MS/MS, and we found that AD‐FSK covalently modified the peptide (residues 355–369) at glutamine (Q) 355 and alanine (A) 356 (Figure 3H). As the previous description, AD‐FSK possessed similar biological activity with FSK (Figure 2B–D) and FSK decreased TGM2 pulled down by AD‐FSK for competition (Figure 2G), indicating both FSK and AD‐FSK interacted with TGM2 in the same sites.

To map the binding reactions between FSK and TGM2, we docked FSK into the structure of TGM2 (PDB code: 1KV3)^[^ ^20^ ^]^ around residues 355–369. In the docking 3D model (Figure 3I), two hydrogen bonds were formed between FSK and asparagine (N) 229 (2.8 Å) or threonine (T) 368 (3.3 Å). Notably, both of these residues were located in the catalytic core domain. Furthermore, N229 and T368 were mutated to alanine (A). SPR analysis demonstrated that FSK showed a weaker ability to bind to TGM2 N229A (*K*_D_ = 26 µm, Figure 3J) or TGM2 T368A (*K*_D_ = 64 µm, Figure 3K) than TGM2 wild type (*K*_D_ = 3.1 µm). Therefore, these results further support that FSK binds to TGM2 by forming hydrogen bonds with N229 and T368 in the catalytic core domain.

### 2.4 FSK Activates TGM2 via Allosterically Inducing an “Open” Conformation

**Figure 4:**
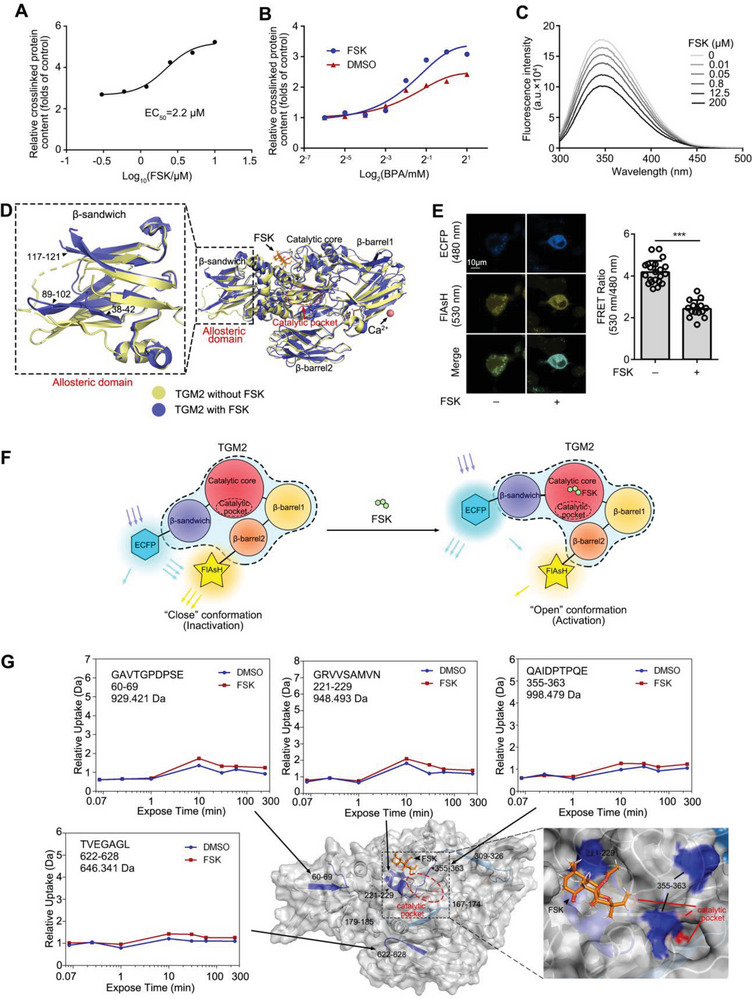
FSK activates TGM2 via allosterically inducing an “open” conformation. A) FSK activated transglutaminase activity of TGM2 (EC_50_ = 2.2 µm). B) Transglutaminase activity of TGM2 in different concentration substrate BPA. C) Tryptophan fluorescence scanning analysis of TGM2. D) 3D model of TGM2 allosteric regulation by molecular dynamics simulation. The allosteric N‐terminal *β*‐sandwich domain is emphasized by the dotted rectangle. E) FlAsH‐based FRET assay in cells with or without FSK treatment (*n* = 14 cells). F) Schematic diagram of FRET assay and FSK‐induced TGM2 “open” conformation. E) FSK allosterically regulated TGM2 conformation by HDXMS. Peptides with increased deuterium uptake ratio after FSK treatment are highlighted in blue.

To explore the effect of FSK on TGM2 biological activity, we tested the transglutaminase activity of TGM2.^[^ ^21^ ^]^ As a result, we found that FSK obviously increased the content of labeled proteins for transamidation reaction catalyzed by TGM2 with an EC_50_ of 2.2 µm (**Figure** **4**A, Figure S6A, Supporting Information). Moreover, we tested the transglutaminase activity in different concentration substrate 5‐(biotinamido) pentylamine (BPA). We found the substrate‐activity curve was sigmate and FSK treatment made the curve move to the upper left, suggesting that FSK probably induced TGM2 activation via an allosteric mechanism (Figure 4B, Figure S7B, Supporting Information). Next, to confirm the potential allosteric effect of FSK on TGM2 structure, we performed tryptophan fluorescence scanning of TGM2. We observed that TGM2 fluorescence intensity was significantly decreased by FSK (Figure 4C), indicating an obvious conformational change. Additionally, molecular dynamics simulation (MDS) analysis was conducted on both “open” and “close” TGM2 confirmations. We found that FSK induced the conformation change of TGM2 “close” structure (PDB:1kv3) change (Gibbs free energy was −8.65 kcal mol^−1^) in the N‐terminal *β*‐sandwich domain, particularly in residues 38–42, 89–102 and 117–121. Meanwhile, the N‐terminal *β*‐sandwich domain moved away from the transglutaminase catalytic pocket to promote the formation of more exposed domains (Figure 4D). It is well known that the catalytic pocket of TGM2, comprising D358, H335, C277,^[^ ^22^ ^]^ is buried inside in “close” conformation and exposed to the surface in “open” conformation.^[^ ^23^ ^]^ Then, MDS analysis between FSK and “open” conformation (PDB:2q3z) (Gibbs free energy was −16.38 kcal mol^−1^) indicated that FSK did not further induce obvious conformation change (Figure S6F, Supporting Information). Next, SPR analysis showed that the *K*_D_ of FSK to “open” conformation (86 µm) is higher than that (0.72 µm) in “close” conformation (Figure S6G, Supporting Information). Therefore, these observations indicated that FSK was inclined to bind to TGM2 in “close” conformation as a transition state, thereby dynamically inducing the “close” conformation change into the “open” conformation of TGM2.

To verify the existence of the “close‐to‐open” conformation change, we designed a TGM2 fluorescence resonance energy transfer (FRET) plasmid containing enhanced cyan fluorescent protein (ECFP) on the N‐terminal *β*‐sandwich domain and a 12‐amino acid peptide (FLNCCPGCCMEP) on the C‐terminal *β*‐barrel2 domain. Fluorescein arsenical hairpin binder (FlAsH) can specifically bind on the 12‐amino acid peptide. We found that FSK treatment significantly reduced the FRET ratio of FlAsH to ECFP (Figure 4E), indicating that the distance between N‐terminal *β*‐sandwich domain and C‐terminal *β*‐barrel2 domain was significantly increased by FSK. Furthermore, hydrogen deuterium exchange mass spectrometry (HDXMS) was performed to support the allosteric regulation mechanism. As a result, FSK treatment increased the hydrogen/deuterium exchange levels of seven specific peptides (peptides 60–69, 167–174, 179–185, 221–229, 309–326, 355–363, and 622–628, Figure 4G, Figure S6C‐E, Supporting Information). Noteworthy, peptides 221–229 and 355–363 are closed to the catalytic pocket (red residue), and they showed higher H/D exchange rates upon FSK treatment (Figure 4G), indicating that TGM2 catalytic pocket was induced to be exposed. Besides, peptides 60–69 and 622–628 with higher H/D rates also suggested these areas were exposed on TGM2 surface during allosteric progress (Figure 4G).

### 2.5 Effects of TGM2 Knockdown on FSK‐Induced MC3T3‐E1 Cell Differentiation

**Figure 5:**
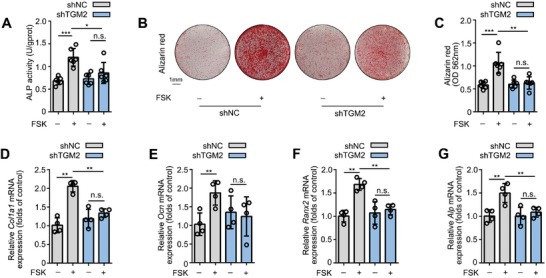
Effects of TGM2 knockdown on FSK‐induced MC3T3‐E1 cell differentiation. A) Knockdown of TGM2 reversed the FSK‐promoted ALP activity in MC3T3‐E1 cells. B,C) Knockdown of TGM2 reduced the promotion of FSK on mineralization in MC3T3‐E1 cells. D–G) TGM2 knockdown inhibited the promotion of FSK on the expression of osteogenesis marker genes including *Col1a1*, *Ocn*, *Ranx2* and *Alp* in MC3T3‐E1 cells. Data are expressed as mean ± SEM for 3–6 individual experiments. ***p < 0.001, **p < 0.01, *p < 0.05.

To test the potential role of TGM2 in MC3T3‐E1 cell differentiation by FSK, TGM2 was knocked down by shTGM2 lentivirus transfection (Figure S7H, Supporting Information). In MC3T3‐E1 cells, TGM2 knockdown significantly inhibited ALP activity that was promoted by FSK (**Figure** **5**A). Moreover, Alizarin red staining demonstrated that the area of FSK‐mediated mineralization nodes and the absorbance of the alizarin red dye extracted from the mineralization nodes were not significantly changed in TGM2 knockdown MC3T3‐E1 cells (Figure 5B,C). Meanwhile, TGM2 knockdown inhibited FSK‐induced *Col1a1*, *Ocn*, *Ranx2*, and *Alp* genes expression (Figure 5D–G, Supporting Information). Thus, these results confirm that FSK accelerates osteogenic differentiation by targeting TGM2.

Interestingly, it was reported that FSK activated adenylate cyclase at picomolar range to increase cAMP content,^[^ ^24^ ^]^ indicating that FSK may promote osteoblast differentiation by targeting adenylate cyclase.^[^ ^25^ ^]^ Here, CETSA analysis suggested that FSK showed weak binding capacity with adenylate cyclase than TGM2 in MC3T3 cells (Figure S7I, Supporting Information). Meanwhile, no adenylate cyclase signal was detected in photocrosslinking analysis in MC3T3 cells (Figure 2E), indicating that FSK may show varying targets‐binding abilities in different situations. To further explore the role of adenylate cyclase in FSK‐mediated osteoblast differentiation, MC3T3‐E1 cells were treated with adenylate cyclase inhibitor SQ22536^[^ ^26^ ^]^ to block adenylate cyclase function. We found that FSK still significantly promoted ALP activity (Figure S7A, Supporting Information), mineralization nodes number (Figure S7B,C, Supporting Information), and the expression of osteoblast differentiation genes (Figure S7D–G, Supporting Information) in the presence of SQ22536, indicating that adenylate cyclase was only partly involved in FSK‐mediated osteoblast differentiation.

### 2.6 Mitochondrial Homeostasis Contributes to TGM2‐Mediated MC3T3‐E1 Cell Differentiation

**Figure 6:**
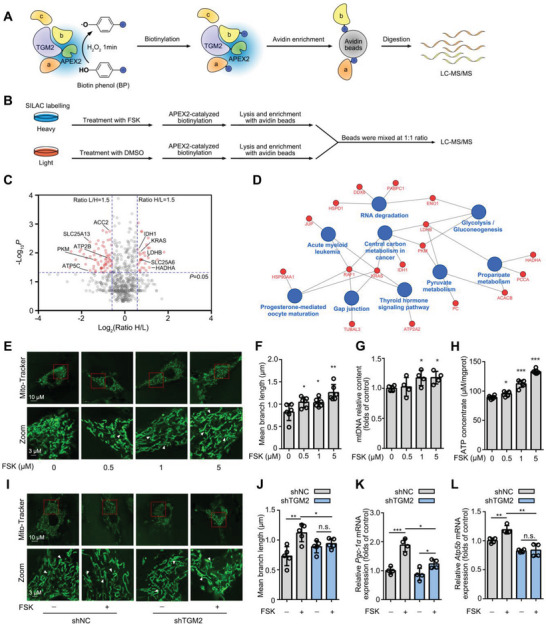
Mitochondrial homeostasis contributes to TGM2‐mediated MC3T3‐E1 cell differentiation. A) Schematic diagram of the APEX2‐mediated proximity labeling strategy. B) Flow chart of TGM2‐interacting protein identification. C) Volcano plots of TGM2‐interacting proteins by FSK treatment. Red dots represent the proteins with significant differences. D) Network diagram of pathway enrichment analysis of TGM2‐interacting proteins. E,F) FSK promoted mitochondrial branch length in MC3T3‐E1 cells. White arrows indicate the representative region. G) FSK upregulated the mtDNA content in MC3T3‐E1 cells. H) FSK increased ATP content in MC3T3‐E1 cells. I,J) Knockdown of TGM2 reversed the FSK‐mediated increase in mitochondria branch length. White arrows indicate the representative region. K,L) The relative expression of the mitochondrial biomarker genes *Pgc‐1α* and *Atp5b* with TGM2 knockdown. Data are expressed as mean ± SEM for 3–6 individual experiments. ***p < 0.001, **p < 0.01, *p < 0.05.

To investigate the potential biological mechanism of TGM2‐mediated MC3T3‐E1 cell differentiation, ascorbate peroxidase 2 (APEX2)^[^ ^27^ ^]^ was performed to evaluate the interaction proteins with TGM2 (**Figure** **6**A). SILAC‐labeled cells were transfected with TGM2‐APEX2 fusion plasmid. After FSK treatment, proximity‐dependent biotin labeling of proteins via APEX2 was enriched with streptavidin beads, followed by LC‒MS/MS analysis (Figure 6B). Then, we identified a total of 101 TGM2‐interacting proteins in volcano plots (Figure 6C). Red dots represented the interacting proteins upregulated (high H/L ratio) or downregulated (low H/L ratio) by FSK treatment with significant differences. Particularly, we observed that an amount of TGM2 interaction proteins were involved in mitochondrial signaling to regulate energy metabolism. Moreover, KEGG analysis (Figure 6D) indicated that a variety of pathways including propanoate metabolism, central carbon metabolism, pyruvate metabolism, thyroid hormone, gap junction and RNA degradation were markedly altered by FSK treatment. Thus, we hypothesized that FSK activated TGM2 to promote energy metabolism in mitochondria to drive osteogenesis. To this end, we detected the mitochondrial morphology in MC3T3‐E1 cells by Mito‐Tracker staining. As a result, The mean mitochondrial branches became longer from 0.822 to 1.258 µm (*p* < 0.05) in the presence of 5 µm FSK in MC3T3‐E1 cells (Figure 6E,F). Moreover, Rt‐PCR analysis revealed that the mitochondrial DNA (mtDNA) content in MC3T3‐E1 cells was significantly upregulated by FSK treatment (Figure 6G). Meanwhile. FSK induced ATP production in MC3T3‐E1 cells (Figure 6H). Furthermore, the mitochondrial morphology was detected with TGM2 knockdown. As shown in Figure 6I,J, TGM2 knockdown obviously blocked the mitochondrial morphology change mediated by FSK in MC3T3‐E1 cells. Meanwhile, TGM2 knockdown also inhibited FSK‐upregulated mitochondrial biomarker gene expression including *Pgc‐1α* ^[^ ^28^ ^]^ and *Atp5b* ^[^ ^29^ ^]^(Figure 6K,L). Besides, FSK promoted TGM2 to colocalize with mitochondria (Figure S8A, Supporting Information). Collectively, these results indicate that FSK promotes energy metabolism for osteogenic differentiation by upregulating mitochondrial function in MC3T3‐E1 cells.

To explore the promotion effect of adenylate cyclase on mitochondrial homeostasis, adenylate cyclase inhibitor SQ22536 was treated in MC3T3‐E1 cells. It was found that FSK still significantly promoted the mean mitochondrial branch length (Figure S8D,E, Supporting Information), content of mtDNA (Figure S8G, Supporting Information), relative expression of mitochondrial marker genes (Figure S8B,C, Supporting Information), and ATP concentration (Figure S8F, Supporting Information) in the presence of SQ22536, indicating that FSK may promote mitochondrial energy metabolism via an independent mechanism rather than cAMP pathway.

### 2.7 FSK Alleviates Osteoporosis in the Ovariectomy Mice Model

**Figure 7:**
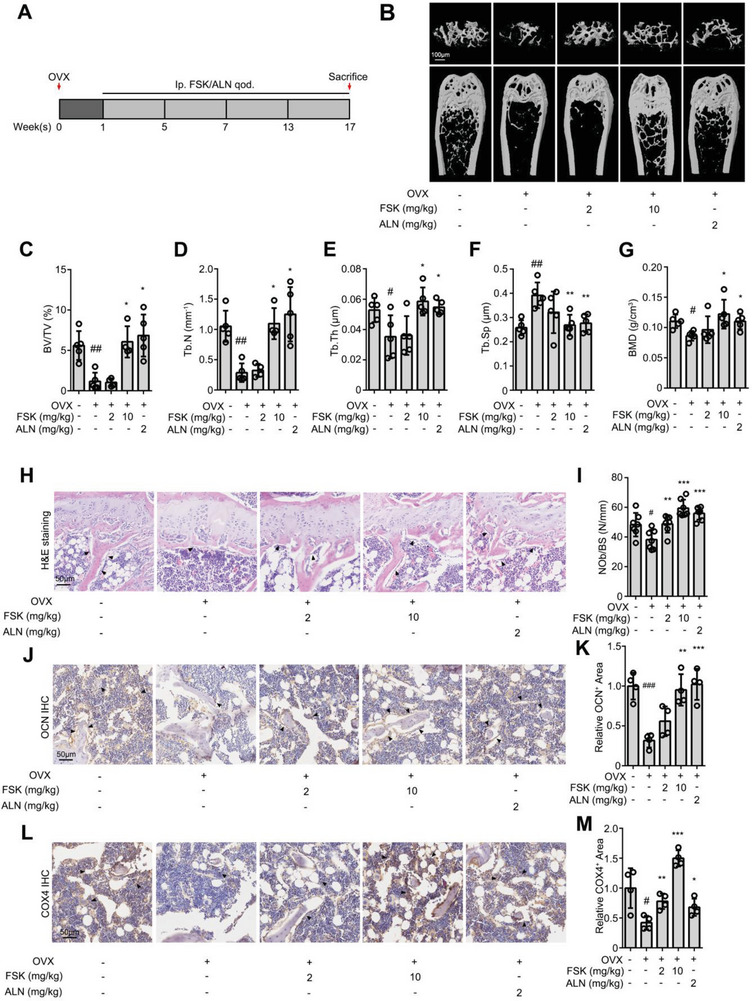
FSK alleviates osteoporosis in the ovariectomy mice model. A) Intraperitoneal injection FSK or ALN once every 2 days for 16 weeks after ovariectomy. B) Representative micro‐CT and reconstructed 3D images of femora in ovariectomy osteoporosis mice on week 16. C–G) Corresponding measurements of bone mineral density (BMD), relative bone volume (BV/TV), trabecular number (Tb.N), trabecular thickness (Tb.Th), and trabecular separation (Tb.Sp). H,I) Representative H&E staining images showing the osteoblast number. Black arrows indicate osteoblasts. J,K) Representative OCN immunohistochemical images showing the number of mature osteoblasts. Black arrows indicate OCN positive region. L,M) Representative COX4 immunohistochemical images showing cell energy metabolism. Black arrows indicate COX4 positive region. Data are expressed as mean ± SEM for 4–6 individual experiments. ***p < 0.001, **p < 0.01, *p < 0.05, ^#^ *p* < 0.05, ^##^ *p* < 0.01, ^###^ *p* < 0.001.

To reveal that FSK may promote the osteoblastic differentiation in vivo for translational medicine study, we established the ovariectomy‐induced osteoporosis mice model (OVX), which were treated by intraperitoneal injection once 2 days with FSK and alendronate sodium (ALN), a clinical anti‐osteoporosis agent for 16 weeks (**Figure** **7**A). The dose of FSK and ALN were designed according to previous reports.^[^ ^30^ ^]^

By micro‐CT analyzing, we found that bone mineral density, relative bone volume, trabecular number, and trabecular thickness significantly decreased and trabecular separation increased than sham group, suggesting a typical osteoporosis symptom. Importantly, FSK significantly improved bone mineral density, relative bone volume, trabecular number, trabecular thickness, and reduced trabecular separation (Figure 7B–G). Furthermore, Micro‐CT analysis showed a thinner cortical bone in OVX mice than sham group, and FSK administration alleviated OVX‐induced bone loss (Figure S9A,B, Supporting Information). In addition, H&E staining of femora slides showed that FSK reversed the decrease of osteoblast number in bone surface (Figure 7H,I). Moreover, the osteocalcin (OCN) level, a marker of mature osteoblast, was significantly increased upon FSK treatment (Figure 7J,K), suggesting that FSK improved osteoporosis by promoting osteoblast differentiation.

To explore bone formation in mice, the procollagen I N‐terminal propeptide (PINP) concentration in serum was detected by ELISA. As a result, the PINP concentration decreased significantly of OVX mice than sham group (*p* < 0.001) and was effectively recovered treated with FSK (*p* < 0.001) (Figure S9G, Supporting Information). Similarly, FSK treatment significantly increased the PINP positive region in OVX mice, indicating obvious promotion effect of FSK for bone formation (Figure S9C, Supporting Information). Indicating obvious promotion effect of FSK for bone formation. Meanwhile, cathepsin K (CTSK) positive osteoclasts were increased in OVX mice than sham group but there was no significant difference on the expression of CTSK with FSK treatment in the bone marrow of OVX mice (Figure S9D, Supporting Information), which demonstrated that osteoblasts but not osteoclasts were responsible for the anti‐osteoporosis effect of FSK.

We also observed that a mitochondria marker cytochrome C oxidase 4 (COX4) in bone marrow noticeably increased with FSK treatment (Figure 7L,M). These results suggested that FSK promoted mitochondrial energy metabolism in bone marrow for osteoblast differentiation. Furthermore, FSK treatment significantly promoted the expression of mitofusin 2 (MFN2) and citrate synthase (CS) in bone marrow, indicating FSK improved energy metabolism by inducing mitochondrial fusion and enhancing tricarboxylic acid cycle. (Figure S10A,B, Supporting Information). Moreover, no obvious change on p‐CREB expression with FSK treatment, indicating that FSK may tend to mainly mediate TGM2 function for activating downstream mitochondria‐dependent anabolic response rather than cAMP signal (Figure S10C, Supporting Information).

Of note, although it was reported that TGM2 activation was associated with the development of cancer,^[^ ^31^ ^]^ celiac disease^[^ ^32^ ^]^ and neurodegenerative diseases,^[^ ^33^ ^]^ no obvious tumors, diarrhea, and cognitive behavioral impairments were found in mice during FSK administration. Moreover, H&E staining of livers and kidneys in mice were performed and there was no obvious pathological change in cytomorphology (Figure S9E,F, Supporting Information). These results confirmed that FSK‐mediated TGM2 activation did not produce serious side effects.

## 3 Discussion

Osteoporosis is the most prevalent bone disease. Osteoblast malfunction induces bone formation abnormalities, resulting in low bone mass and fragile fractures in bone diseases.^[^ ^34^ ^]^ Therefore, the development of novel drugs that induce osteoblast differentiation and maturation may help to maintain bone homeostasis and control skeletal development, formation and remodeling.^[^ ^35^ ^]^ However, there is a serious lack of effective therapeutic targets for this pathological process. In this study, through a chemical genetic study using FSK as a chemical probe, we identified a unique allosteric site on TGM2 for inducing its enzyme activity that promotes osteoblast differentiation.

Currently, TGM2 inhibitors have been widely reported to show anticancer effects such as GK921^[^ ^36^ ^]^ However, the specific TGM2 activator is still unknown. Here, we revealed FSK as a selective TGM2 activator to promote osteogenesis. Mechanistically, FSK exhibited an allosteric effect on TGM2 by inducing a previously unexpected “open” conformation. We speculated that FSK‐mediated “open” conformation may form an efficient substrate‐recognition platform on TGM2, thereby leading to accelerated capture and process of substrate proteins. Of note, we further elucidated a pharmacological binding pocket of FSK at a cave with N229 and T368, which was located in a saddle‐like surface. To our knowledge, this is the first reported allosteric activation site on TGM2 for inducing osteoblast differentiation, and can be used for further lead compound screening to develop novel TGM2 activators.

As a natural‐derived small molecule, FSK may interact multiple target proteins to exert its biological activities. Therefore, FSK may potentially promote osteoblast differentiation via multiple cellular targets such as TGM2 and adenylate cyclase. In the present study, we preferred to exploring new druggable targets that contribute osteoblast differentiation via using FSK as a chemical probe. Here, TGM2 was identified as a crucial cellular target of FSK for osteoblast differentiation, which provides a potential direction to deplore new drug candidate for osteoporosis.

Mitochondria play a crucial role in bone metabolism and bone diseases by regulating the biology of bone tissue cells.^[^ ^37^ ^]^ Increasing evidence shows that mitochondria provide a place for ATP production to drive osteoblast differentiation.^[^ ^34^, ^38^ ^]^ Here, we showed that TGM2 knockdown significantly reversed FSK‐induced mitochondrial fusion as well as ATP production, indicating that TGM2 played a potential role in mitochondrial function maintenance. Moreover, we employed APEX2‐based proteomics to identify several mitochondrial dynamics or energy metabolism‐associated functional proteins including HADHA, SLC25A6 and ATP5C. Previous studies have demonstrated that these proteins mainly contribute to mitochondrial *β*‐oxidation,^[^ ^39^ ^]^ ATP/ADP transfer^[^ ^40^ ^]^ and ATP synthesis,^[^ ^41^ ^]^ thereby indicating a potential biological relationship between TGM2 and mitochondrial energy synthesis. Therefore, pharmacological targeting of the TGM2/mitochondrial signaling pathway may serve as a promising strategy to lead compound discovery for bone metabolism regulation.

## 4 Conclusion 

In this study, we identified TGM2 as the direct cellular target of FSK to induce osteoblast differentiation. In particular, we revealed an allosteric activation site in the catalytic core of TGM2. FSK induced an unexpected “open” confirmation of the N‐terminal *β*‐sandwich domain, which accelerated the substrate protein crosslinks. We also systematically explored the interactome of TGM2 for interrogating the mitochondrial energy generation mechanism in osteoblast differentiation and proved anti‐osteoporosis effect of FSK in vivo. Therefore, these findings provide a fresh perspective for exploring TGM2 biology in osteoblast differentiation and enable new opportunities for innovative drug development for osteoporosis.

## 5 Experimental section

Supporting Materials and Methods, Supporting Data are provided in Supporting Information.

## Conflict of Interest

The authors declare no conflict of interest.

## Supporting information

Supporting Information

## Data Availability

The data that support the findings of this study are available from the corresponding author upon reasonable request.
