# Supplementary material for: Allosteric Activation of Transglutaminase 2 via Inducing an “Open” Conformation for Osteoblast Differentiation
Source: Adv Sci (Weinh). 2023 Apr 23;10(18):2206533. doi: 10.1002/advs.202206533 (PMC10288273; doi:10.1002/advs.202206533)
Supplement: Supplementary file 1 — Supporting Information [file ADVS-10-2206533-s001.pdf]

ADVANCED SCIENCE
Open Access

Supporting Information

for *Adv. Sci.*, DOI 10.1002/advs.202206533

Allosteric Activation of Transglutaminase 2 via Inducing an "Open" Conformation for Osteoblast Differentiation

*Zhuo Yang, Xiao-Wen Zhang, Fang-Fang Zhuo, Ting-Ting Liu, Qian-Wei Luo, Yong-Zhe Zheng, Ling Li, Heng Yang, Yi-Chi Zhang, Yan-Hang Wang, Dan Liu, Peng-Fei Tu and Ke-Wu Zeng**

Supporting Information

Allosteric activation of transglutaminase 2 via inducing an "open" conformation for osteoblast differentiation

Zhuo Yang[1], Xiao-Wen Zhang[1], Fang-Fang Zhuo[1], Ting-Ting Liu[1], Qian-Wei Luo[1], Yong-Zhe Zheng[1], Ling Li[1], Heng Yang[1], Yi-Chi Zhang[1], Yan-Hang Wang[1], Dan Liu[2], Peng-Fei Tu[1], Ke-Wu Zeng[1, *]

[1]*State Key Laboratory of Natural and Biomimetic Drugs, School of Pharmaceutical Sciences, Peking University, Beijing 100191, China.*

[2]*Proteomics Laboratory, Medical and Healthy Analytical Center, Peking University Health Science Center, Beijing 100191, China.*

[*]Corresponding author. State Key Laboratory of Natural and Biomimetic Drugs, School of Pharmaceutical Sciences, Peking University, Beijing 100191, China.

Email address: ZKW@bjmu.edu.cn (K. Zeng)

Experimental section

Chemical and materials

Forskolin ($C_{22}H_{34}O_7$, relative molecular mass, 410.51) was purchased from Aladdin (Shanghai, China) and affirmed by ^{1}HNMR and ^{13}CNMR data. 1-(3-Dimethylaminopropyl)-3-ethylcarbodiimide (EDC), *N,N*-diisopropylethylamine (DIPEA), 4-dimethylaminopyridine (DMAP), tris(2-carboxyethyl) phosphine (TCEP), tris[(1-benzyl-1*H*-1,2,3-triazol-4-yl) methyl] amine (TBTA) and 3-(3-(but-3-yn-1-yl)-3*H*-diazirin-3-yl) propanoic acid were purchased from Sigma-Aldrich (St. Louis, MO, USA). Biotin-PEG3-azide and 5-(biotinamido) pentylamine (BPA) were purchased from AAT Bioquest (Mercury Drive Sunnyvale, CA, USA). Biotin-phenol was purchased from Topscience (Shanghai, China). Ascorbic acid, β-phosphoglyceride, Trolox and sodium azide were purchased from Aladdin (Shanghai, China). Alizarin red solution was purchased from Leagene Biotechnology (Beijing, China). Anti-transglutaminase 2 antibody (ab2386) was purchased from Abcam (Cambridge, UK). Streptavidin-HRP (3999S) was purchased from CST Cell Signaling Technology (Beverly, MA, USA). Anti-β-actin antibody (66009-1-Ig), anti-COX4 antibody (11242-1-AP) and anti-OCN antibody (16157-1-AP) were purchased from Proteintech (Chicago, IL, USA).

Cell culture

Mouse embryo pre-osteoblast MC3T3-E1 and human embryonic kidney 293 cells (HEK293) were provided by Peking Union Medical College Cell bank (Beijing, China). MC3T3-E1 cells were maintained in Alpha Minimum Essential Medium (α-MEM) supplemented with 10% fetal bovine serum and 1% streptomycin/penicillin. HEK293 cells were maintained in high glucose Dulbecco’s Modified Eagle Medium (DMEM) supplemented with 10% fetal bovine serum and 1% streptomycin/penicillin. The cells were incubated at 37 °C with 5% CO_2 in culture incubator.

Animals

C57bl/6 female mouse were purchased from Department of Laboratory Animal Science in

Peking University Health Science Center and kept at 25±1 °C under 12/12h light/dark cycle. Experimental procedures were approved (LA2022506) by the Ethical Institutional Animal Care and Use Committee of Peking University, Beijing, China.

Alizarin red staining

MC3T3-E1 cells were treated with FSK (0.5, 1, 5 μM) to induce osteogenesis differentiation with medium containing 5 mM β-glycerol phosphate disodium salt and 50 μg/mL ascorbic acid for 21 days. Then, the cells were fixed with 4% paraformaldehyde and incubated in Alizarin red solution (pH 4.2) for 10 min at room temperature. The mineralization nodes were dyed red and detected by SMZ745 stereoscopic microscope (Nikon, Tokyo, Japan). The alizarin red complex was dissolved with 10% cetylpyridinium chloride (CPC, Adamas, China) and the absorbance of the solution was quantificationally measured at 562 nm with a spectrophotometer (BioTek, USA).

ALP activity assay

MC3T3-E1 cells were treated with FSK (0.5, 1, 5 μM) to induce osteogenesis differentiation for 5 days. Then, the cells were lysed with 1% triton in PBS and ALP activity was detected with ALP assay kit (Nanjing Jiancheng Bioengineering Institute, Nanjing, Jiangsu, China). Briefly, 30 μL reagent 1, 30 μL reagent 2 and 15 μL lysate were mixed and incubated at 37 °C for 15 min. Next, 200 μL reagent 3 was added and the optical densities (ODs) were detected at 520 nm using microplate reader (Austria GmbH 5082 Grodlg, Tecan, Männedorf, Switzerland).

The synthesis of FSK molecular probe (AD-FSK)

3-(3-(But-3-yn-1-yl)-3*H*-diazirin-3-yl) propanoic acid 20.4 mg, forskolin 108.0 mg, EDC 102.6 mg, DMAP 8.7 mg and 100 μL DIPEA were dissolved in dry dichloromethanol (5 mL) under argon environment. The reaction mixture was stirred for 24 h at room temperature in darkness. The AD-FSK was purified by column chromatography on silica gel eluted with dichloromethane: methanol (75:1). AD-FSK was obtained 12.6 mg as yellow solid. The structure of AD-FSK was verified by ^{1}H-NMR, ^{13}C-NMR and high-resolution mass

spectrometry. ^{1}H NMR (500 MHz, $CDCl_3$) δ 5.89 (dd, *J* =, 10.6, 1.3 Hz, 1H), 5.55 (brs, 1H), 5.47 (d, *J* = 1.3 Hz, 1H), 5.32-5.21 (m, 1H), 4.96 (d, *J* = 10.6 Hz, 1H), 4.50-4.42 (m, 2H), 3.13 (dd, *J* = 16.6, 1.3 Hz, 1H), 2.43 (dd, *J* = 16.6, 1.3 Hz, 1H), 2.18 (d, *J* = 7.4, 4H), 2.14-2.12 (m, 1H), 2.08-2.01 (m, 1H), 1.99 (qd, *J* = 7.4, 4.6 Hz, 4H), 1.84 (s, 2H), 1.77 (t, *J* = 7.4 Hz, 2H), 1.70 (s, 3H), 1.69 (s, 1H), 1.67-1.53 (m, 4H), 1.35-1.28 (m, 3H), 1.26 (s, 3H), 1.19-1.10 (m, 1H), 1.04 (s, 3H), 0.06 (d, *J* = 1.3 Hz, 1H).^{13}C NMR (125 MHz, $CDCl_3$) δ 205.00, 170.08, 169.78, 146.01, 110.56, 82.73, 82.12, 81.93, 77.42, 77.16, 76.91, 76.33, 75.64, 69.84, 69.43, 48.66, 43.94, 43.33, 36.93, 34.33, 33.05, 32.41, 31.42, 29.15, 27.74, 24.43, 23.78, 23.52, 21.31, 19.90, 13.40. MS: m/z calcd for $C_{30}H_{42}N_2O_8$ $[M+H]^+$: 559.30, found: 559.32.(Figure S1-3)

Immunoblots

MC3T3-E1 cells were lysed in NP-40 Lysis Buffer (Beyotime, Shanghai, China) supplemented 1% protease inhibitor. The concentration of protein was tested with BCA method (TransGen, Beijing, China). Proteins were separated by 10-15% SDS-PAGE and electro-transferred onto PVDF membranes. The membranes were blocked with 5% nonfat-dried milk for 1 h at room temperature and incubated with primary antibodies overnight at 4 °C. Subsequently, the membranes were washed and incubated with secondary antibody conjugated HRP for 2 h at room temperature. Finally, immunoblots were visualized by a Tanon 5200 Imaging Analysis System (Tanon, Shanghai, China).

Click chemistry for target identification

MC3T3-E1 cells were incubated with AD-FSK (50 μM) for 4 h and exposed to 365 nm UV light for 10 min. Cells were lysed in NP-40 Lysis Buffer (Beyotime, Shanghai, China) supplemented 1% protease inhibitor and adjusted to a consistent concentration and volume with NP-40 Lysis Buffer. Cells lysates containing 1 mg proteins were successively mixed with 3 μL Biotin-PEG_3-N_3 (20 mM), 11.6 μL TCEP (14.4 mg/mL), and 1.75 μL TBTA (34 mM). The click reaction was initiated by 11.6 μL $CuSO_4$ (50 mM). The reaction was incubated for 1 h at room temperature. The protein pellets were collected by 6000 g centrifugation and were washed with cold methanol twice. Then, the samples were heated in 1.2% SDS at 85 °C for

5 min. Then streptavidin beads (30 μL) were mixed with samples and incubated at room temperature for 3 h. The biotinylated proteins captured on streptavidin beads were digested to peptides with trypsin. These peptides were analyzed by LC-MS/MS (NanoLC-LTQ Velos pro MS, Thermo, Waltham, MA, USA).

These peptides were separated in C18 reversed-phase column. The elution buffer A was 0.1% formic acid in water and the elution buffer B was 0.1% formic acid in ACN. The procedure for gradient eluting were 2%-40% B for 70 min; 40%-95% B for 5 min; 95%B for 20 min. The eluent was introduced to the MS at 300 nL/min. MS parameters were listed as follows: scan spectra, 350 to 2000 m/z; maximum IT, 50 ms; HCD Collision Energy, 35%; resolution, 60000; Target FDR for PSMs, 0.01. MS data were analyzed with Proteome Discoverer (version 1.4) software with SEQUEST (Thermo, Waltham, MA, USA).

Cellular thermal shift assay (CETSA)

MC3T3-E1 cells were treated with FSK (50 μM) or DMSO for 2 h at 37 °C. Cells were harvested in PBS containing 1% protease inhibitor and divided into ten groups. The samples were heated for 2 min respectively at gradient temperatures (37-55 °C) with T100 Thermal Cycler (Bio-Rad, Hercules, CA, USA). 20 μL kinase buffer (CST, Beverly, MA, USA) was added into the samples and repeated freeze-thaw in liquid nitrogen was performed to obtain cell lysates. The cell lysates were analyzed by immunoblot with anti-TGM2 antibody.

Drug affinity responsive target stability (DARTS)

MC3T3-E1 cells were lysed with NP-40 Lysis Buffer (Beyotime, Shanghai, China). Lysates were diluted with 10×TNC buffer (50 mM NaCl, 10 mM $CaCl_2$, 50 mM Tris-HCl, pH 8.0) to the same final volume and incubated with FSK (50, 100, 200, 400 μM) at 4 °C for 1 h, followed by addition of 5 μg/mL pronase (Solarbio, Beijing, China) for 20 min in 1×TNC buffer at room temperature. Reactions were terminated by adding SDS loading buffer, followed by TGM2 detection through immunoblot with anti-TGM2 antibody.

TGM2 expression and purification

TGM2 was subcloned into pET-28a vector to create an N-terminal His tag in TGM2.

Escherichia coli Bl21 (DE3) cells (Solarbio, Beijing, China) were transformed with the vector and monoclonal bacterial colony was inoculated 500 mL LB medium (10 g/L peptone, 10 g/L NaCl and 5 g/L yeast extract). The bacteria were grown at 37 °C in 200 rpm until OD_{600} reached 0.6. Then, 1 mM isopropyl β-D-thiogalactoside (IPTG) was added at 16 °C in 120 rpm for 24 h, and the cells were harvested by 4000 rpm centrifugation. Cell pellets were lysed by sonication in 30 mL binding buffer (20 mM HEPES, 250 mM NaCl, 10 mM imidazole). Cell debris was removed by 12000 rpm centrifugation and the supernatant was loaded onto Ni-NTA column (Thermo, Waltham, MA, USA), which was washed by 100 mL binding buffer. After 10 min, the column was washed with 100 mL washing buffer (20 mM HEPES, 250 mM NaCl, 50 mM imidazole) to remove impurities. The TGM2 protein was eluted with elution buffer (20 mM HEPES, 250 mM NaCl, 250 mM imidazole). Proteins were concentrated by centrifugal filtration (Amicon Ultra-15, Millipore, MA, USA) with a molecular weight cutoff of 10 kDa. The protein was loaded onto a molecular sieve chromatography and eluted with PBS. Purity of TGM2 was tested by SDS/PAGE with coomassie brilliant blue staining.

Surface plasmon resonance (SPR) assay

The ability of FSK to bind TGM2 was analyzed by Biacore 8K system (GE Healthcare, Uppsala, Sweden). The recombinant TGM2 protein (50 μg/mL) was immobilized on carboxymethylation 5 (GE Healthcare, Uppsala, Sweden) sensor chip with amine coupling reaction. A gradient concentration (0.78 to 200 μM) of FSK in PBS (containing 5% DMSO) were injected into the sensor chip. For the ability of FSK to bind TGM2 with "open" conformation, a gradient concentration (0.78 to 50 μM) of FSK in 20 mM Tris-HCl buffer (pH 7.5) containing 150 mM NaCl and 10 mM $CaCl_2$ were injected into the sensor chip. SPR data were analyzed by Biacore evaluation software (GE Healthcare, Uppsala, Sweden).

Real time PCR (Rt-PCR) analysis for gene expression

MC3T3-E1 cells were treated with FSK (0.5, 1, 5 μM) for Total RNA was extracted from cells by MolPure Cell/Tissue Total RNA Kit (Yeasen Biotechnology, Shanghai, China). Rt-PCR analyses were performed in Mx3005P Real-Time QPCR System (Agilent, Palo Alto, CA,

USA) with Hieff qPCR SYBR Green Master Mix (Yeasen Biotechnology, Shanghai, China). Primers used to detect osteogenesis markers and mitochondria makers are listed as Supplementary Table 1.

In vitro transglutaminase activity assay

MC3T3-E1 cells were lysed by NP-40 Lysis Buffer (Beyotime, Shanghai, China) and centrifuged to collect total proteins. Recombinant TGM2 protein (100 μg/mL) was incubated with FSK (0.78, 1.56, 3.12, 6.25, 12.5, 25 μM) for 1 h. Then, 10 μL TGM2-FSK complex, 10 μL cell lysate and 180 μL reaction buffer (2 mM BPA, 40 mM $CaCl_2$ and 40 mM dithiothreitol in Tri-HCl buffer) were mixed. The mixture was incubated at 37 °C for 20 min, and the reaction was terminated by boiling in SDS loading buffer, followed by biotinylated proteins detection through immunoblot with streptavidin-HRP. The gray value was calculated by Image J (version 1.48) software.

Identification of FSK-binding peptides on TGM2

TGM2 protein was diluted with TNC buffer to 300 μg/mL and incubated with FSK (50 μM) or equal DMSO on ice for 1 h. Then, the samples were digested with 5 μg/mL pronase (Solarbio, Beijing, China) for 20 min in 1×TNC buffer at 37 °C, and the reaction was terminated by SDS loading buffer. Finally, SDS-PAGE was conducted to separate the samples to determine degradation product. The degradation protein bands were excised and analyzed with LC-MS/MS.

Identification of FSK-binding sites on TGM2

TGM2 protein (100 μg/mL) was incubated with AD-FSK (50 μM) or equal DMSO for 1 h on ice. Then, the samples were exposed to 365 nm UV light for 10 min on ice. The samples were mixed with SDS loading buffer and heated at 98 °C for 10 min. Subsequently, the samples were separated with SDS-PAGE and the main protein band was excised. The proteins were digested with trypsin and analyzed with LC-MS/MS.

TGM2 knockdown with shTGM2 lentivirus

The lentivirus containing shTGM2 was established from Hanbio, Shanghai, China. The lentivirus was transfected into MC3T3-E1 cells with polybrene (8 μg/mL) for 24 h. The cells were screened by the addition of puromycin (10 μg/mL) to establish the stable TGM2 knockdown cells.

TGM2-interaction proteins discovery with APEX2

TGM2-APEX2 plasmid (30 μg) was transfected into MC3T3-E1 cells for 48 h. Then, 500 μM biotin-phenol was incubated for 4 h at 37 °C. 1 mM H_2O_2 was added for 1 min. The cells were washed with quenching buffer (PBS containing 10 mM sodium azide, 10 mM sodium ascorbate, and 5 mM Trolox) three times and centrifuged at 2000 rpm for 2 min. After that, the cells were lysed in RIPA buffer (Beyotime, Shanghai, China) supplemented with protease inhibitors and centrifuged at 12000 rpm for 15 min to remove precipitate. The streptavidin beads were subsequently added to equal concentration proteins and incubated for 2 h at 4 °C. Finally, the samples were sequentially washed with RIPA buffer, 1 M KCl, 0.1 M Na_2CO_3, 2 M urea in 0.1 M Tris-HCl (pH 7.0) buffer and RIPA buffer, followed by digestion with trypsin and analysis with LC-MS/MS.

Mito-Tracker staining assay

MC3T3-E1 cells were treated with FSK (0.5, 1, 5 μM) for 48 h. 100 nM Mito-Tracker Green solution (Beyotime, Shanghai, China) was added to the cells and incubated for 20 min. Images were obtained by laser confocal microscopy (100×) (LSM880, Zeiss, Oberkochen, Germany) with 490 nm exciting wavelength and 516 nm emission wavelength. The images were quantificationally analyzed by Image J (version 1.48) with MiNA 2.0 functional plug for calculating the mitochondrial mean branch length. Each group was evaluated by 6 original images. Statistical analysis was conducted by T-test.

Hydrogen-deuterium exchange mass spectrometry (HDXMS) analysis

TGM2 protein (1 mg/mL) was incubated with FSK (50 μM) or equivalent DMSO in 4 °C for 30 min. The protein was equilibrated in D_2O buffer in room temperature and quenched with 37.5% hydrochloric acid at indicated times. Samples were digested by Waters ENZYMATE BEH

pepsin column. Then the peptides were captured on a VanGuard Pre-Column trap, eluted with 15% acetonitrile and separated using an ACQUITY UPLC BEH C18 column. Relative deuterium levels of all peptides were calculated by subtracting the mass of undeuterated control sample from that of the deuterium-labeled sample.

FlAsH-based FRET

HEK293T cells were cultured in 6 cm dishes to transfect FRET plasmids with Lipofectamine 2000. 48 hours later, cells were digested and planted in 24-well confocal plates. The 5 μM FSK was treated for 2 hours after adherence. Then cells were washed three times with HBSS and treated with 250 nM FlAsH-EDT2 in 37 °C for 20 min. Whereafter, cells were washed three times with 250 μM EDT in HBSS to remove non-specific adsorption and maintained with clean HBSS. Fluorescence images were scanned with laser confocal microscopy (LSM880, Zeiss, Oberkochen, Germany). ECFP was excited at 405 nm, and FlAsH was excited at 514 nm directly. Emission of ECFP and FlAsH was measured at 480 ± 20 nm and 530 ± 20 nm, respectively. FRET ratio was calculated as the ratio of F530 to F480. Fluorescence signals were digitalized using Zen software.

Ovariectomy-induced osteoporosis model

Six-week-old C57bl/6 female mice were anesthetized with isoflurane and shaved on dorsum. A dorsal midline incision was made to exteriorize the ovary. Then the fallopian tube was ligated and the ovary was removed and the wound was sutured. The same process was repeated on the other side. Sham operations were performed in the same way without ligating fallopian tube and removing ovaries. One week after the surgery, the FSK or ALN dissolved in normal saline containing 5 %DMSO and 5% PEG 300 was treated with intraperitoneal injection every other day for 16 weeks.

Micro-CT analysis

The femora were fixed in 4% paraformaldehyde at room temperature. The distal femurs were scanned with Micro-CT image system (SkyScan 1176, Bruker, Germany). Scanning parameters were following, distinguishability 9 μm, voltage 50 kV, electricity 400 μA.

Microstructure of bone trabeculae parameters were analyzed with CTAn software.

Bone sectioning and immunohistochemistry analysis

After sacrifice, the femora were fixed in 4% paraformaldehyde at room temperature for 24 h. Then the femora were decalcified in 10% EDTA (pH=7.5) for 21d. The tissues were dehydrated and embedded in paraffin. Then 4.5 μm-think sections were cut with rotary microtome (RM215, Leica, Germany). The dewaxed sections were eliminated endogenous peroxidase activity and blocked with goat serum. Then sections were incubated in the anti-COX4 and anti-OCN primary antibodies in 4 °C overnight and incubated in secondary antibody for 30 min and visualized with Diaminobenzidine. Sections were scanned with Digital pathology scanner (WS-10, Wisleap, China).

Statistical analysis

Statistical analysis was performed with one-way ANOVAs by using GraphPad Prism 6.0. The experimental results were demonstrated as mean ± SD. $P < 0.05$ was considered to indicate statistical significance.

Supplementary Table 1. Primer pairs for Rt-PCR

Gene	Forward Primer Sequence (5'->3')	Reverse Primer Sequence (5'->3')
Ocn	CACTCCTCGCCCTATTGGC	CCCTCCTGCTTGGACACAAAG
Col1a1	GAGGGCCAAGACGAAGACATC	CAGATCACGTCATCGCACAAC
Ranx2	GCCACCTTTACCTACACCCC	ACTCTGGCTTTGGGAAGAGC
Alp	AACCCAGACACAAGCATTCC	GCCTTTGAGGTTTTTGGTCA
Pgc1-α	CCCTGCCATTGTTAAGACC	TGCTGCTGTTCCTGTTTTC
Atp5b	TTCAGGGGCACCAATCAAAATTC	CAACCTTTATCCCAGTCACCAGA

Results

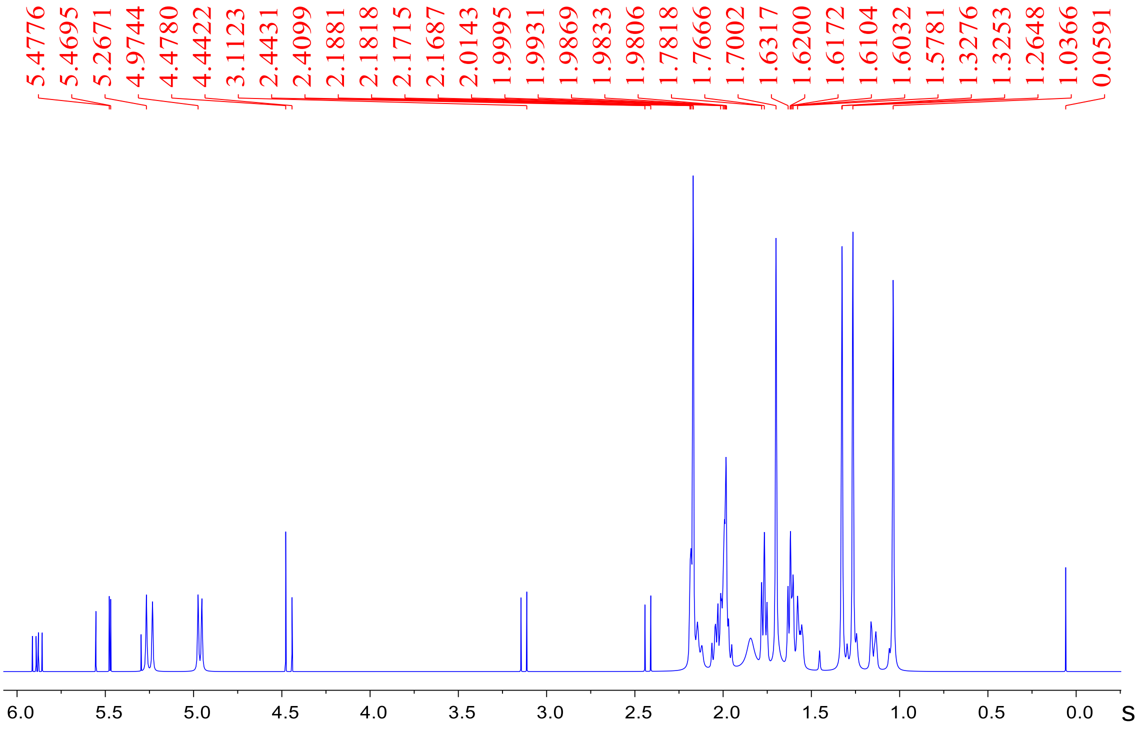

Figure S1. ^{1}H NMR spectrum of AD-FSK.

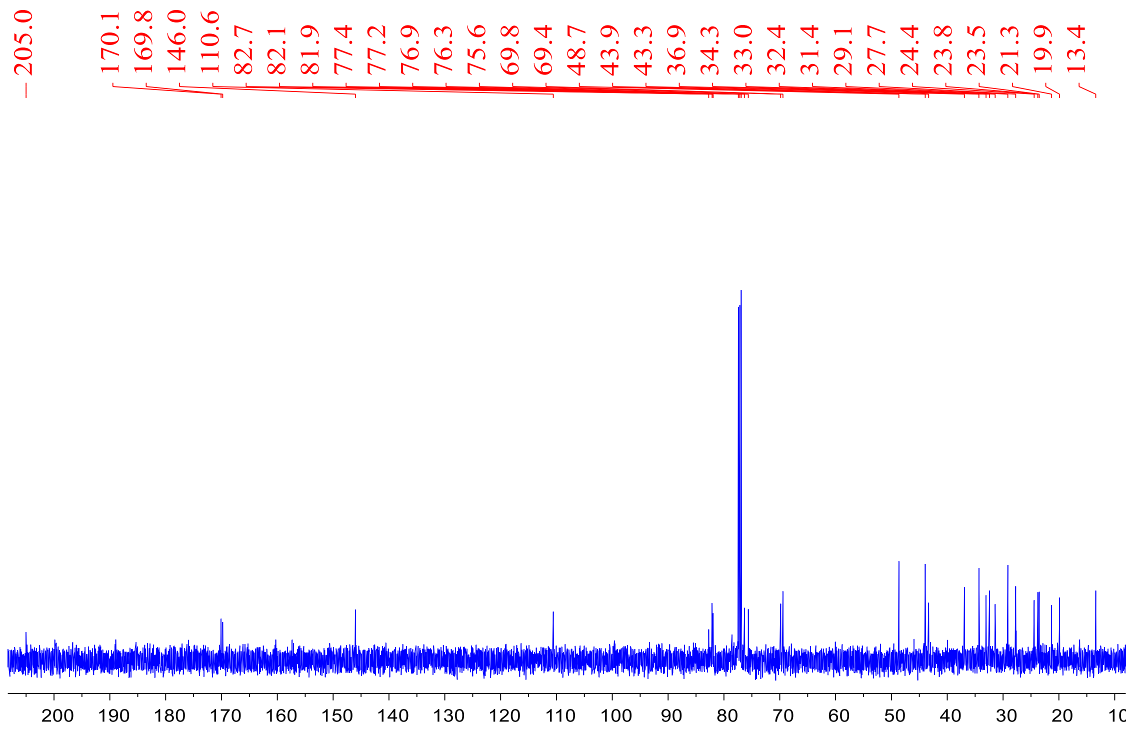

Figure S2. ^{13}C NMR spectrum of AD-FSK.

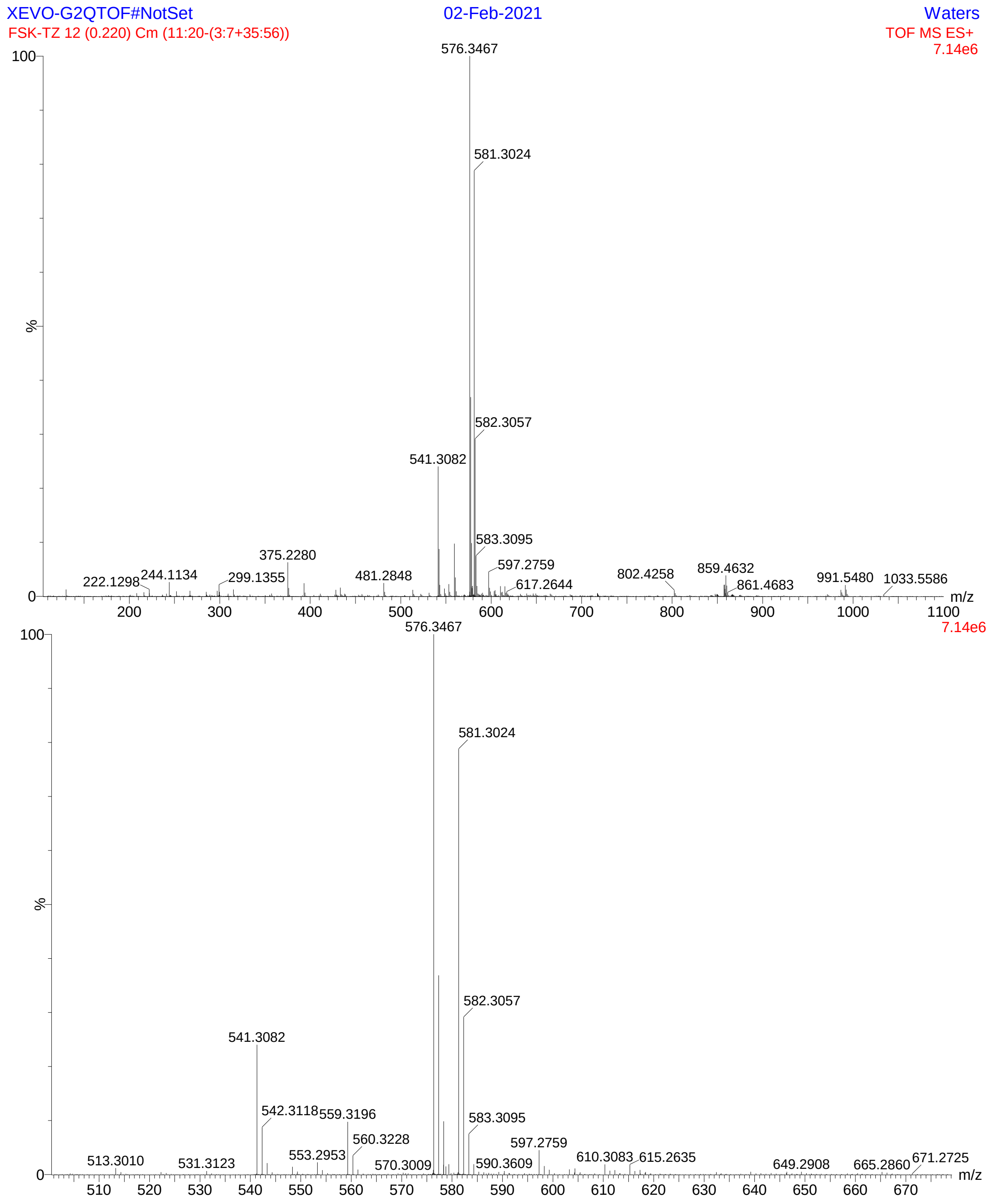

Figure S3. MS of AD-FSK.

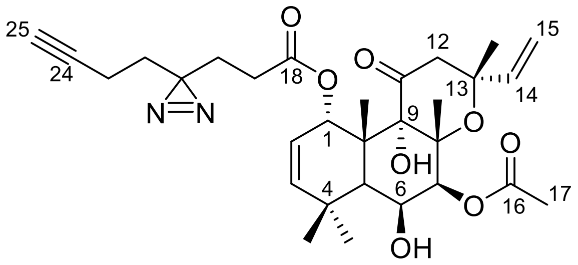

Figure S4. Chemical structure of AD-FSK.

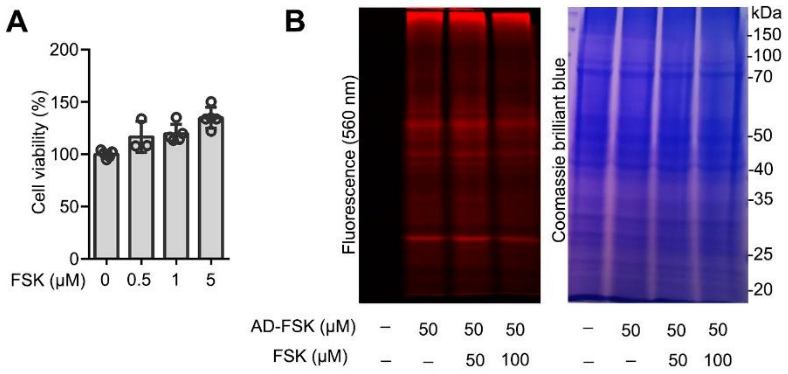

Figure S5. (A) Cell viability was in MC3T3-E1 cells with FSK treatment.

(B) The fluorescence intensity of bands labeled with AD-FSK was competed by FSK.

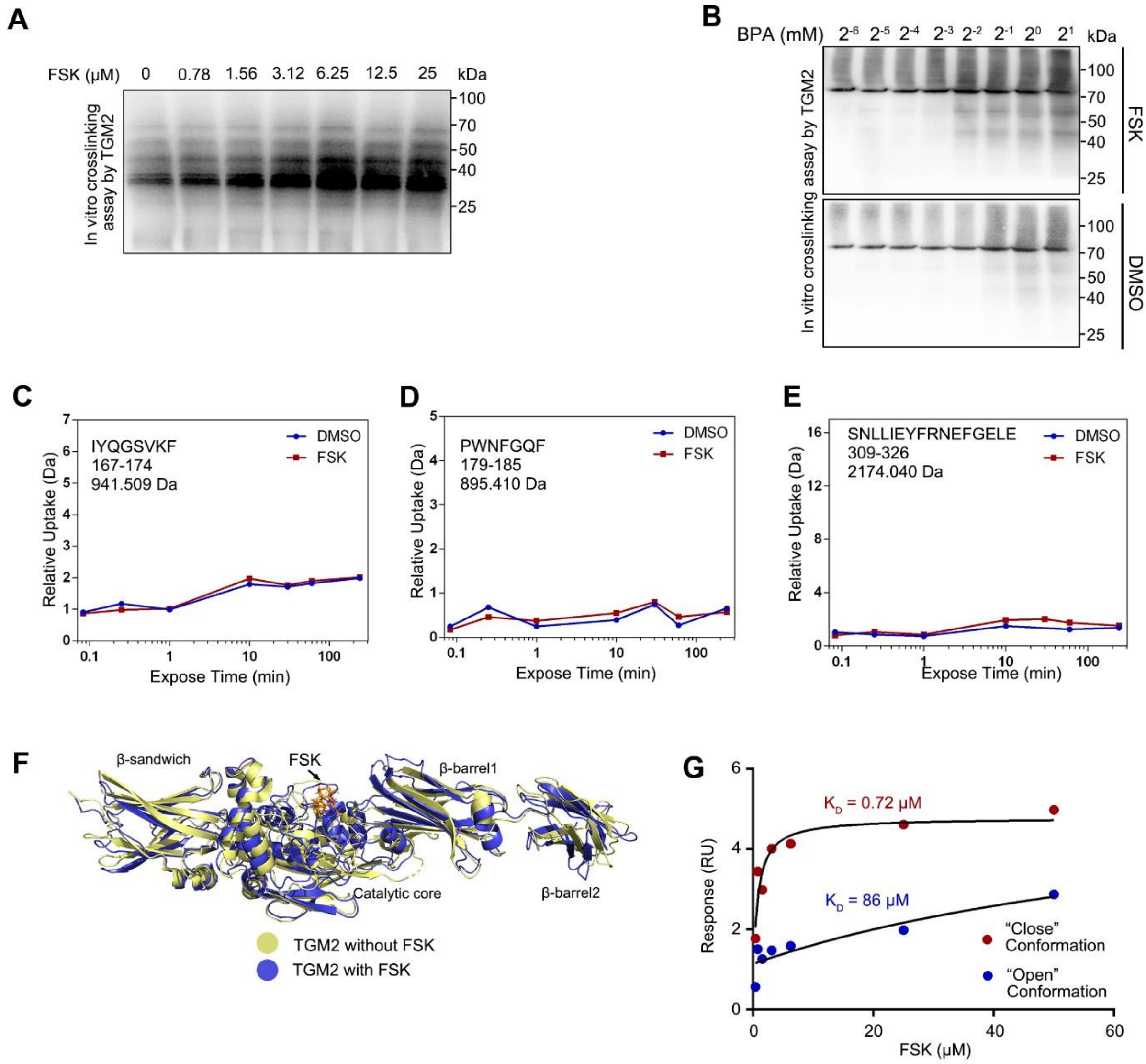

Figure S6. (A-B) The content of TGM2 crosslinking proteins with was detected by immunoblot.

(C-E) H/D exchange rates of peptides 167-174, 179-185, 309-326, and 355-363 suggested the allosteric regulation round the TGM2 catalytic pocket.

(F) Molecular dynamics simulation of FSK and "Open" conformation TGM2 (PDB: 2q3z).

(G) SPR analysis of FSK-binding to TGM2 with different conformations.

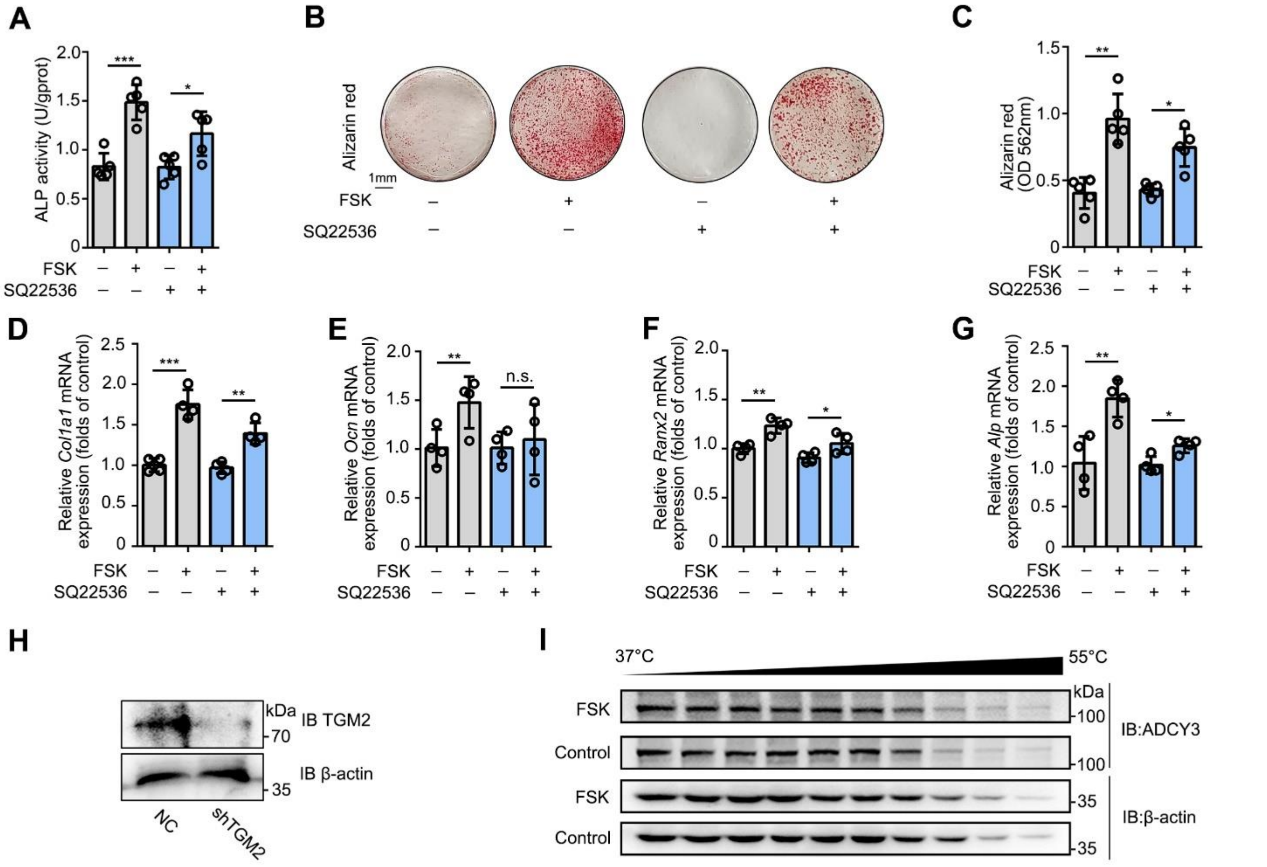

Figure S7. (A) FSK promoted ALP activity upon SQ22536 treatment in MC3T3-E1 cells.

(B-C) FSK increased the number of mineralization nodes upon SQ22536 treatment in MC3T3-E1 cells.

(D-G) FSK up-regulated the expression of osteoblast differentiation genes upon SQ22536 treatment in MC3T3-E1 cells.

(H) TGM2 was knocked down by shTGM2 lentivirus transfection.

(I) FSK hardly promoted resistance of ADCY3 to different temperature gradients (CETSA).

Data are expressed as mean ± SEM for 4-6 individual experiments. ***$P < 0.001$, **$P < 0.01$, *$P < 0.05$.

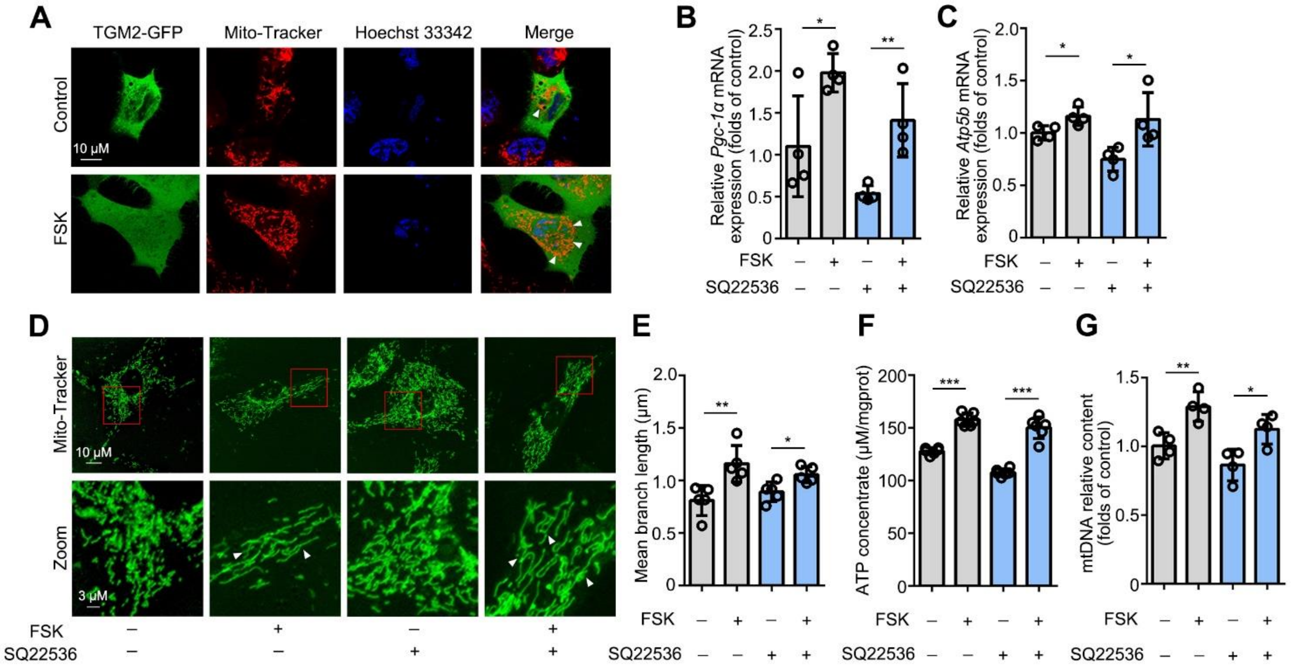

Figure S8. (A) FSK promoted TGM2 (green) to colocalize with mitochondria (red). White arrows indicate the representative region.

(B-C) The relative expression of the mitochondrial biomarker genes *Pgc-1α* and *Atp5b* upon SQ22536 treatment.

(D-E) FSK promoted mitochondrial branch length in MC3T3-E1 cells upon SQ22536 treatment. White arrows indicate the representative region.

(F) FSK increased ATP content upon SQ22536 treatment in MC3T3-E1 cells.

(G) FSK upregulated the mtDNA content upon SQ22536 treatment in MC3T3-E1 cells.

Data are expressed as mean ± SEM for 4-6 individual experiments. ****P* < 0.001, ***P* < 0.01, **P* < 0.05.

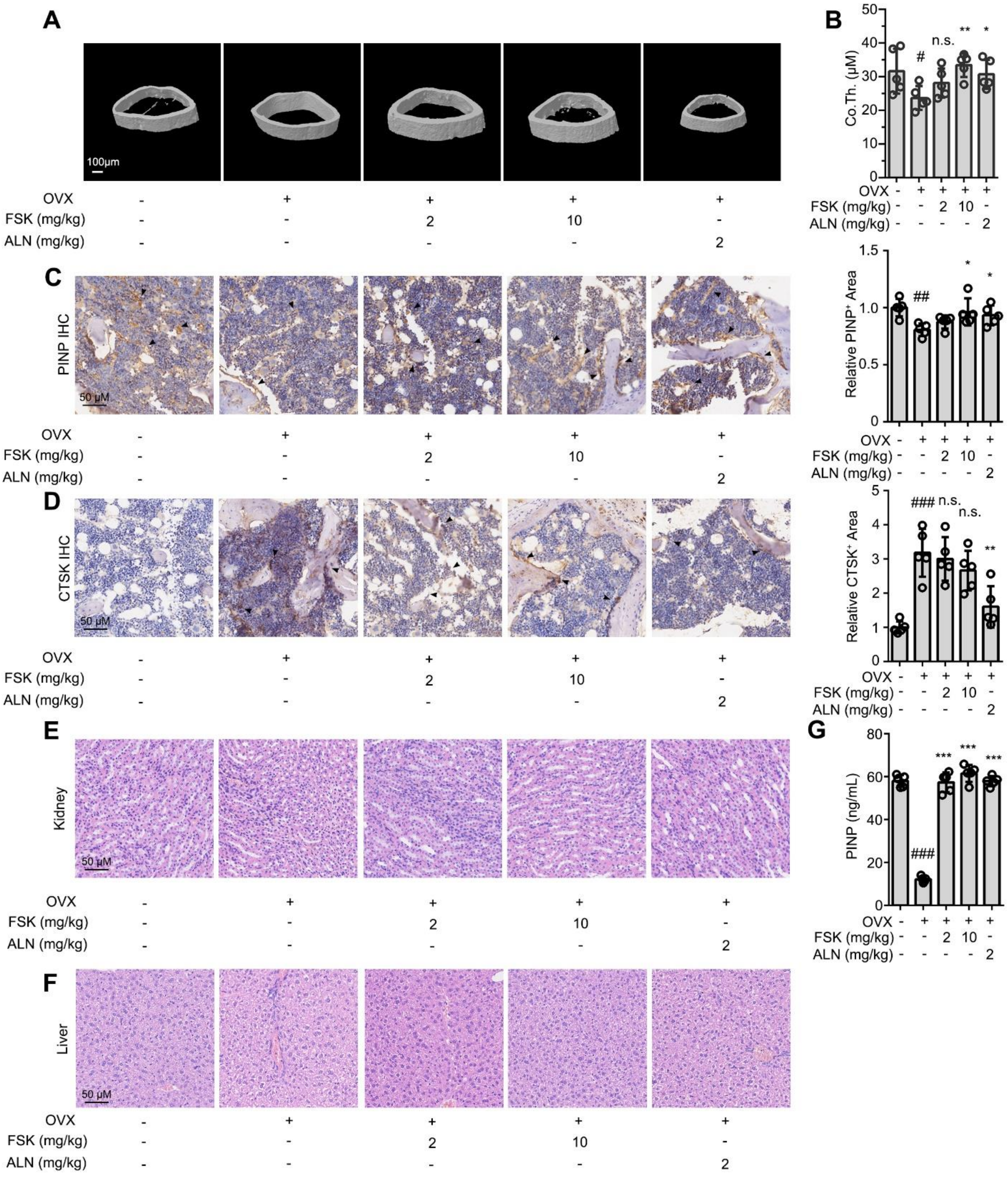

Figure S9. (A) Representative micro-CT and reconstructed 3D images of cortical bone in femora of OVX mice.

(B) FSK increased cortical thickness (Co.Th.) in femora of OVX mice.

(C) Representative PINP IHC images and quantitative analysis showing bone formation. Black arrows indicate the PINP positive region.

(D) Representative CTSK IHC images and quantitative analysis showing bone resorption.

Black arrows indicate the CTSK positive region.

(E) Representative H&E staining images showing the kidneys in mice.

(F) Representative H&E staining images showing the livers in mice.

(G) FSK increased PINP concentration in OVX mice serum.

Data are expressed as mean ± SEM for 4-6 individual experiments. ***$P < 0.001$, **$P < 0.01$, *$P < 0.05$, $^{\#}P<0.05$, $^{\#\#}P<0.01$, $^{\#\#\#}P<0.001$.

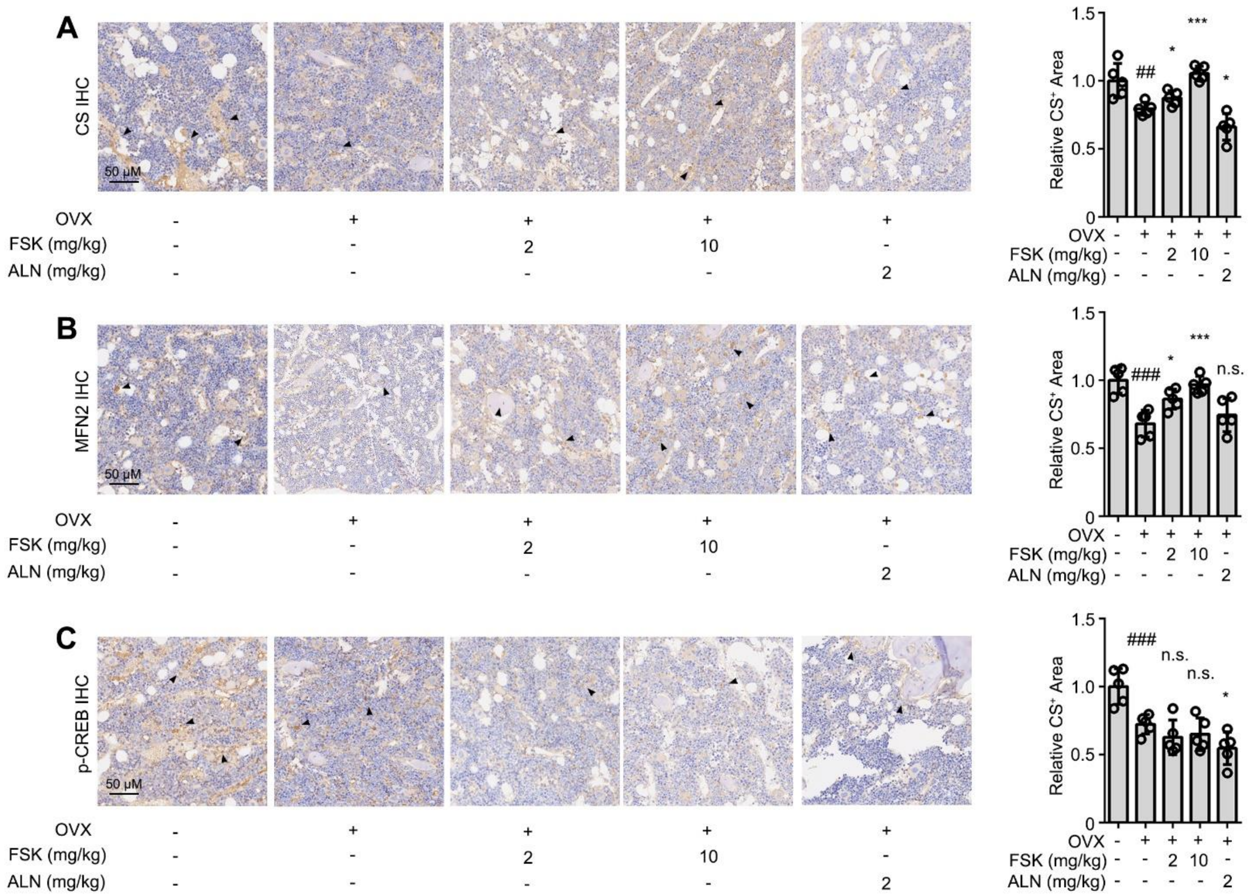

Figure S10. (A-C) Representative images and quantitative analysis of CS (A), MFN2 (B), and p-CREB (C) in OVX mouse femur sections by IHC analysis. Black arrows indicate the positive region.

Data are expressed as mean ± SEM for 4-6 individual experiments. ****P* < 0.001, ***P* < 0.01, **P* < 0.05, $^{\#}P$<0.05, $^{\#\#}P$<0.01, $^{\#\#\#}P$<0.001.
